# CapsID: a web-based tool for developing parsimonious sets of CAPS molecular markers for genotyping

**DOI:** 10.1186/1471-2156-7-27

**Published:** 2006-05-10

**Authors:** Jonathan Taylor, Nicholas J Provart

**Affiliations:** 1 Department of Computer Science, University of Toronto, 40 St George Street, Toronto, ON M5S 2E4, Canada; 2 Centre for the Analysis of Genome Evolution and Function, Department of Cell and Systems Biology, University of Toronto, 25 Willcocks Street, Toronto, ON M5S 3B2, Canada

## Abstract

**Background:**

Genotyping may be carried out by a number of different methods including direct sequencing and polymorphism analysis. For a number of reasons, PCR-based polymorphism analysis may be desirable, owing to the fact that only small amounts of genetic material are required, and that the costs are low. One popular and cheap method for detecting polymorphisms is by using cleaved amplified polymorphic sequence, or CAPS, molecular markers. These are also known as PCR-RFLP markers.

**Results:**

We have developed a program, called CapsID, that identifies snip-SNPs (single nucleotide polymorphisms that alter restriction endonuclease cut sites) within a set or sets of reference sequences, designs PCR primers around these, and then suggests the most parsimonious combination of markers for genotyping any individual who is not a member of the reference set. The output page includes biologist-friendly features, such as images of virtual gels to assist in genotyping efforts. CapsID is freely available at .

**Conclusion:**

CapsID is a tool that can rapidly provide minimal sets of CAPS markers for molecular identification purposes for any biologist working in genetics, community genetics, plant and animal breeding, forensics and other fields.

## Background

DNA sequences from different varieties or accessions of a given species are becoming available through a number of sequencing projects. Single nucleotide polymorphisms (SNPs), together with insertion/deletions (InDels), are the most common type of polymorphism in the genomes studied so far. Large sets of predicted SNPs are publicly available for the human genome (SNP Consortium, ), and for some genetic model organisms, including *Caenorhabditis elegans *[1], *Drosophila melanogaster *[2], and *Arabidopsis thaliana *[3]. In addition, the generation of short stretches of DNA sequence information for non-model organisms, e.g. from ribosomal sequences, is becoming increasingly economical. Approximately 30–40% of SNPs alter restriction endonuclease recognition sites and these are commonly referred to as snip-SNPs [1]. Restriction enzyme digestion pattern polymorphism may be used to create cleaved amplified polymorphic sequence (CAPS) markers (also known as PCR-RFLP markers), which are codominant molecular markers that amplify a short genomic sequence around the polymorphic endonuclease restriction site [4]. They are easily detected by agarose gel electrophoresis. CAPS are thus affordable and practical for genotyping in positional or map-based cloning projects [4-6] and in molecular identification studies, where direct sequence-based identification is not necessary or practical.

With the increasing availability of parallel genomic sequences from different varieties or accessions of genetic model species and/or the use of genetic fingerprinting methods for forensic and other work, there is a need for a web-based, user-friendly program that facilitates snip-SNP-based CAPS marker design. Currently there is no free software tool available for rapid detection of the restriction site polymorphisms in aligned sequences. Some related applications are for conceptually different markers (SNAPER [7]), are not freely available for bench scientists through a web interface (autoSNP [8]), or have a very limited sequence size input and no sequence alignment option (dCAPS Finder [9,10]), or only identify potential CAPS markers [11] and do not automate the process of selecting the most useful and parsimonious set of CAPS markers for genotyping.

To overcome these limitations we created CapsID, a web-based program intended for bench scientists, which identifies differential endonuclease restriction sites in multiple sequence alignments. CapsID then generates the appropriate primers to use, and selects the smallest number of CAPS markers to unambiguously identify a member of a set. Primers are generated automatically, if desired, with the Primer3 [12] program, and in addition virtual gel pictures for each species (sequence) are generated.

## Implementation

Each CAPS marker splits a set of candidate species or strain sequences into two or more testable species or strain sequence sets. In the following example we assume that there is only one target snip-SNP within the amplified region, so:

*Set*1 = {*Sequences the restriction enzyme targeting the CAPS cuts in*}

*Set*2 = {*Sequences the restriction enzyme targeting the CAPS does not cut in*}

The presence of many possible CAPS markers splits the candidates into many testable sets. Ideally we would like to split *N *candidates into *N *testable sets. Our preference for many small testable sets may be summarized by the equation:

*Cost*(*Sets*) = ∑S∈Sets|S|2
 MathType@MTEF@5@5@+=feaafiart1ev1aaatCvAUfKttLearuWrP9MDH5MBPbIqV92AaeXatLxBI9gBaebbnrfifHhDYfgasaacH8akY=wiFfYdH8Gipec8Eeeu0xXdbba9frFj0=OqFfea0dXdd9vqai=hGuQ8kuc9pgc9s8qqaq=dirpe0xb9q8qiLsFr0=vr0=vr0dc8meaabaqaciaacaGaaeqabaqabeGadaaakeaadaaeqbqaaiabcYha8jabdofatjabcYha8naaCaaaleqabaGaeGOmaidaaaqaaiabdofatjabgIGiolabdofatjabdwgaLjabdsha0jabdohaZbqab0GaeyyeIuoaaaa@3C27@, where |*S*| is the number of species in the set *S*.

This will assign a high cost to few large sets, and a low cost to many small sets. Consider the following example:

Given candidate species sequences = {A, B, C, D} and CAPS = {1,2,3}

If CAPS 1 gives sets {A,B} and {C, D}

and CAPS 2 gives sets {A,B,C} and {D}

and CAPS 3 gives sets {A} and {B,C,D}

then CAPS 1 and 2 give sets {A,B}, {C} and {D}

and CAPS 1 and 3 give sets {A}, {B}, {C,D}

and CAPS 1, 2 and 3 give sets {A}, {B}, {C}, {D}, which is what we want. The cost for each possible combination is determined using the above equation:

CAPS 1: Cost({A,B},{C,D}) = 2^2 ^+ 2^2 ^= 8

CAPS 2: Cost({A,B,C}, {D}) = 3^2 ^+ 1^2 ^= 10

CAPS 3: Cost({A}, {B,C,D}) = 1^2 ^+ 3^2 ^= 10

CAPS 1, 2: Cost({A,B}, {C}, {D}) = 2^2 ^+ 1^2 ^+ 1^2 ^= 6

CAPS 1, 3: Cost({A}, {B}, {C, D}) = 1^2 ^+ 1^2 ^+ 2^2 ^= 6

CAPS 1, 2, 3: Cost({A}, {B}, {C}, {D}) = 1^2 ^+ 1^2 ^+ 1^2 ^+ 1^2 ^= 4

Thus to minimize cost we choose CAPS 1 then 2 then 3 – and not CAPS 2 then 1 then 3, because the cost function progresses from highest to lowest. Algorithmically, for each possible CAPS marker, a cost is assigned based on the size of the sets created. The one with the lowest cost wins. Following this, the remaining potential CAPS markers are examined, in the context of the sets generated by the winner in the first step. Again the winner is chosen based on the smallest cost for the new sets created by the combined action of the 2 CAPS markers. The algorithm proceeds until the number of sets is equal to the number of candidate sequences. If this criterion cannot be met, then the algorithm alerts the user that there are not enough CAPS markers to unambiguously identify the candidates. If there is a tie in terms of the cost function, CapsID offers these to the user as alternate CAPS markers.

CapsID is programmed in Python. Each sequence alignment is parsed using Biopython [13] into either a Bio.Clustalw.ClustalAlignment or Bio.Fasta.FastaAlignment object. Both of these types are subclasses of a more general Alignment class, allowing for both to be handled transparently. The alignments are subsequently processed by the Bio.CAPS module, which we created and have submitted to the Biopython repository [13]. This module makes use of the Bio.Restriction package to computationally "digest" each sequence in the alignment according to the user-specified enzymes. The algorithm keeps track of the sets generated and uses the cost function described to identify the most parsimonious number of CAPS markers. The Bio.CAPS package can also utilize Biopython's Bio.Emboss.Primer module, a wrapper for a primer design program called Primer3 [12], so that primers may be generated using a need_primers call. Finally, we have written a module called gel.spy that uses the Python Imaging Library to generate a virtual gel representation of the fragments on the fly.

## Results and discussion

**Figure 1:**
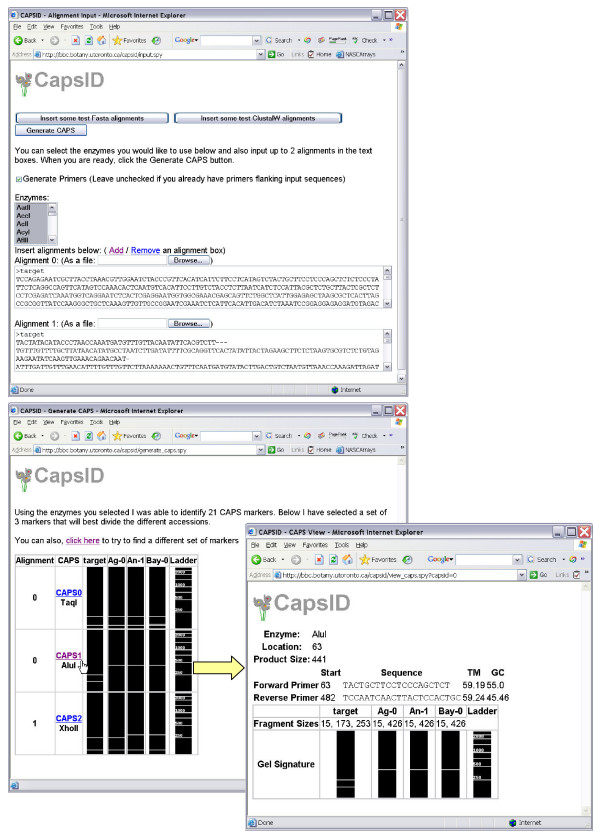
**CapsID Screenshots**. The top panel illustrates the input page of CapsID. Inputs allowed are the desired enzymes to be used for the CAPS markers, and a multiple sequence alignment(s) of sequences from the reference organisms. The bottom panel shows the CapsID output page summarizing the number of potential CAPS markers found, as the most parsimonious set to use to genotype organisms not in the reference set. Clicking on a given CAPS marker results in a page containing 'virtual' gel images for that CAPS marker in each reference organism to be generated, as well as the primers and the exact cut locations of the indicated enzyme.

The sequence input page contains input boxes to paste or upload a set of alignments, as shown in Figure 1 in the top panel. The alignments can be in Fasta or Clustal format. The user can then select which enzymes should be considered for CAPS marker generation.

When the user asks for CAPS markers to be created, many Biopython modules are employed in series to generate a marker set. The alignments are first parsed from their native file format into uniform alignment data structures. These data structures are then computationally analyzed for potential CAPS markers. The program then uses a cost function, explained in the Implementation section, to weight each potential set of CAPS markers, so that an optimal set can be found.

This optimal set is presented to the user with all the information needed to use the CAPS markers. Of note are fields for enzyme, primer sequences and primer location. To aid in marker identification, a pseudo gel is also displayed using the Python Imaging Library, as seen in Figure 1 in the bottom panel. This pseudo gel is an approximation of what should be seen on the gel when a marker is tested. For a given set of sequences there may be more than one equally parsimonious set of CAPS markers that may be used for genotyping, and in this case the user may click through these sets and inspect the resultant pseudo gels for the 'best' set, in terms of fragment size differences.

## Conclusion

In conclusion, CapsID is an easy-to-use web-based tool for generating the smallest set of CAPS markers to genotype an individual. It is expected that this tool will be of great use to biologists working in community genetics, plant and animal breeding, forensics and other fields.

## Availability and requirements

Project name: CapsID

Project home page: 

Operating system: Any web-browser able to display images will work

Programming language: Python

Other requirements: for local installation, several Python modules are necessary

License: GNU GPL

Any restrictions to use by non-academics: none

## Authors' contributions

JT carried out the implementation of the algorithm, designed the cost function to identify the most parsimonious sets, programmed the web interfaced and helped with the manuscript. NJP wrote and edited the manuscript, conceived the study and supervised JT. All authors read and approved the final manuscript.

## References

[B1] Wicks SR, Yeh RT, Gish WR, Waterston RH, Plasterk RH (2001). Rapid gene mapping in Caenorhabditis elegans using a high density polymorphism map. Nat Genet.

[B2] Hoskins RA, Phan AC, Naeemuddin M, Mapa FA, Ruddy DA, Ryan JJ, Young LM, Wells T, Kopczynski C, Ellis MC (2001). Single nucleotide polymorphism markers for genetic mapping in *Drosophila melanogaster*. Genome Res.

[B3] Torjek O, Berger D, Meyer RC, Mussig C, Schmid KJ, Rosleff Sorensen T, Weisshaar B, Mitchell-Olds T, Altmann T (2003). Establishment of a high-efficiency SNP-based framework marker set for Arabidopsis. Plant J.

[B4] Konieczny A, Ausubel FM (1993). A procedure for mapping Arabidopsis mutations using co-dominant ecotype-specific PCR-based markers. Plant J.

[B5] Glazebrook J, Drenkard E, Preuss D, Ausubel FM (1998). Use of cleaved amplified polymorphic sequences (CAPS) as genetic markers in *Arabidopsis thaliana*. Methods Mol Biol.

[B6] Lukowitz W, Gillmor CS, Scheible WR (2000). Positional cloning in Arabidopsis. Why it feels good to have a genome initiative working for you. Plant Physiol.

[B7] Drenkard E, Richter BG, Rozen S, Stutius LM, Angell NA, Mindrinos M, Cho RJ, Oefner PJ, Davis RW, Ausubel FM (2000). A simple procedure for the analysis of single nucleotide polymorphisms facilitates map-based cloning in Arabidopsis. Plant Physiol.

[B8] Barker G, Batley J, O' Sullivan H, Edwards KJ, Edwards D (2003). Redundancy based detection of sequence polymorphisms in expressed sequence tag data using autoSNP. Bioinformatics.

[B9] Neff MM, Neff JD, Chory J, Pepper AE (1998). dCAPS, a simple technique for the genetic analysis of single nucleotide polymorphisms: experimental applications in *Arabidopsis thaliana *genetics. Plant J.

[B10] Neff MM, Turk E, Kalishman M (2002). Web-based primer design for single nucleotide polymorphism analysis. Trends Genet.

[B11] Ilic K, Berleth T, Provart NJ (2004). BlastDigester: a web-based program for efficient CAPS marker design. Trends Genet.

[B12] Rozen S, Skaletsky H, Krawetz S, Misener S (2000). Primer3 on the WWW for general users and for biologist programmers. Bioinformatics Methods and Protocols: Methods in Molecular Biology.

[B13] Biopython Project and Repository. http://www.biopython.org.

